# *PICALM::MLLT10* may indicate a new subgroup of acute leukemias with miscellaneous immunophenotype and poor initial treatment response but showing sensitivity to venetoclax

**DOI:** 10.1002/jha2.922

**Published:** 2024-05-15

**Authors:** Haimin Sun, Yongmei Zhu, Jianfeng Li, Lingling Zhao, Guang Yang, Zeying Yan, Sujiang Zhang

**Affiliations:** ^1^ Department of Hematology, Ruijin Hospital Shanghai Jiao Tong University School of Medicine Shanghai China; ^2^ Shanghai Institute of Hematology State Key Laboratory of Medical Genomics, National Research Center for Translational Medicine at Shanghai, Ruijin Hospital Shanghai Jiao Tong University School of Medicine Shanghai China

**Keywords:** acute leukemia, immunophenotyping, *PICALM::MLLT10*, venetoclax

## Abstract

The *PICALM::MLLT10* fusion gene is a rare but recurrent event in acute leukemia (AL) associated with poor prognosis. It is still confused whether *PICALM::MLLT10* can solely correspond to acute myeloid leukemia (AML) or acute lymphoblastic leukemia (ALL) or acute leukemias of ambiguous lineage (ALAL). Here, we reported a series of *PICALM::MLLT10* positive AL patients with miscellaneous immunophenotype including T‐ALL, ALAL, AML, and B‐ALL, complex karyotype, half of extramedullary disease (EMD), frequently concomitant PHF6 mutation, and poor initial treatment response to standard chemotherapy aiming to different immunophenotype, but showing sensitivity to combining chemotherapy especially integrated with venetoclax, suggesting this fusion gene may indicate a new subgroup of AL. Eighteen *PICALM::MLLT10* positive patients of 533 AL patients (18/533, 3.4%) were identified by RNA sequencing in our center. We found *PICALM::MLLT10* positive AL showing miscellaneous immunophenotype, higher expression of leukemic stemness genes and lower expression of biomarkers of venetoclax resistance, more extramedullary involvement, and especially poor response to conventional induction chemotherapy, but may benefit from venetoclax as well as low‐dose Ara‐C, granulocyte colony‐stimulating factor (G‐CSF), and anthracyclines combination chemotherapy. Sequential hematopoietic stem cell transplantation (HSCT) after chemotherapy combined with venetoclax may further improve long‐term survival in AL patients with complete remission (CR) even measurable residual disease (MRD) positive.

## 1 INTRODUCTION

The *PICALM::MLLT10* fusion gene, generated by chromosome aberration of t(10;11)(p12.3;q14.2), is a rare but recurrent event in acute leukemia (AL), which was identified in about 7% of patients with T‐cell acute lymphoblastic leukemia (T‐ALL) and other leukemia, associated with the upregulation of the proto‐oncogenic *HOXA* and *MEIS1* genes and poor prognosis [1, 2, 3, 4].

The 2022 WHO classification further mentioned that genomic findings such as *PICALM::MLLT10* fusions are enriched in mixed‐phenotype acute leukemias (MPALs), which belong to acute leukemias of ambiguous lineage (ALAL) but need more data [5, 6]. In 2022 European LeukemiaNet (ELN) classification, *PICALM::MLLT10* was identified as acute myeloid leukemia (AML) with other rare recurring translocations but not ALAL [7]. In addition, there is no separate classification for *PICALM::MLLT10* in the new international consensus classification (ICC) classification [8]. Therefore, it is still confused whether *PICALM::MLLT10* can solely correspond to AML or ALL or ALAL [9].

Here, we reported a series of *PICALM::MLLT10* positive AL patients with unique clinical characteristic and treatment response, suggesting this fusion gene may indicate a new subgroup of AL.

## 2 CASE SERIES PRESENTATION

Eighteen *PICALM::MLLT10* positive patients of 533 AL patients (18/533, 3.4%) were identified by RNA sequencing (RNA‐seq) in our center from July 2020 to September 2023, including five female and 13 male, with a median age of 32 years (16–50 years). These patients were newly diagnosed AL according to bone marrow morphology and immunology including six ALAL (6/14), seven T‐ALL [six early T‐cell precursor ALL (ETP‐ALL), one cortical T‐ALL] (7/27), three AML (3/369), one B‐ALL with aberrant expression of myeloid antigen (1/112), and one B/T MPAL (1/11). The mean white blood cell counts of these patients were 7.5 × 10^9^/L (1.65—167 × 10^9^/L), hemoglobin level was 119.5 g/L (ranging from 69 to 165 g/L), and platelet counts were 168.5 × 10^12^/L (5–429 × 10^12^/L) individually, while the median blast cells were 80.6% (15%–94%). It should be mentioned that extramedullary disease (EMD) was found in nine cases (9/18), including mediastinum, tonsil, and skin. In terms of immunotyping, CD7 was identified in all patients (18/18) and CD33 in 77.8% (14/18) patients (Table 1 and Figure S1A–F), suggesting that CD7 may serve as the potential diagnostic marker and immunotherapy target especially in non‐T AL. The major concurrence mutations were *PHF6* mutation (11/18), *JAK3* mutation (5/18), and *SUZ12* mutation (5/18), with less common mutations including *WT1*, *NOTCH1*, *SETD2*, *FAT1*, *ETV6*, *KRAS*, *NRAS*, *RUNX1*, *SF3B1*, *ASXL1*, and so forth. Characteristic cytogenetic abnormality t(10;11) (p12.3;q14.2) was found in 10 cases (10/18) and six of them (6/10) were found to be associated with complex karyotype (Table 1).

**TABLE 1:** Characteristics of *PICALM::MLLT10* positive AL patients.

N	G/A	WBC/HB/PLT (×10^9^/)	EMD	FAB/Blast (%)	Immunophenotype	Gene mutation	Karyotype
1	M/44	8.7/128/133	/	AL/94	ALAL, CD7/33/ 34/DR+	SETD2 M761I, IKZF1 P154fs, EED Y308S, SUZ12 L668P	86–96, XXYY, add(1)(p36)x2, del(2)(q34)×2, add(9)(q34)x2, +5–12mar[cp5]
2	M/40	24/151/429	Y	ALL/83.7	ETP‐ALL, CD7/ 34/5/cyCD3+	NOTCH1 F1592_L1593insDHH, R2313Kfs*25, NOTCH1 W2310S, JAK3 M511I, PHF6 C85*, FAT1 T2369P, FAT1 D2274E, FAT1 A1564T	44–46, XY, t(10;11;1) (p12;q14;p36) [cp5]/46, XY[5]
3	F/34	6.6/133/270	Y	AL/38	ALAL, CD7/33/ 34/71/DR+	EZH2 c.1505+1G>C, ETV6 V345G, KRAS G60V, RUNX1 V452G, KDM6A V1344fs	46, XX[20]
4	M/32	33/97/121	/	AL/93.5	ALAL, CD7/33/11b+	SF3B1 A899P, SETD2 S1572_N1573insGG, ARID5B E576*, ASXL1 Q428fs, PHF6 C297Y	46–49, XY, +6, del(9)(q31), t(10;11) (p12;q14), add (12)(p13), −17, +19, +1–2mar[cp6]/46, XY[4]
5	M/30	15.7/148/60	Y	ALL/71	Cortical T‐ALL, CD7/5/4/8/1a/10/99/cyCD3+	NOTCH1 L1593P, S2450fs, WT1 V376fs, SUZ12 G42fs, FAT1 A1575T	46, XY[20]
6	M/34	5.7/152/330	Y	ALL/48	ETP‐ALL, CD7/ 34/33/5/cyCD3+	SUZ12 R196fs, JAK3 M511I, PHF6 D141fs, Notch1 P1606delinsLSP, Q2403*	46, XY, t(10;11)(p12;q14)[1]/46, XY[17]
7	F/28	36/111/231	/	ALL/70	B/T, CD7/34/cyCD3/19/79a+	FLT3‐TKD S838N, FBXW7 R465C, ARID1 M910T, NF1 c.3496+1G>T, ASXL1 C594*, U2AF1 R35L, BCOR W509*, PHF6 G291V	47–55, XXX, +1, +6, +7, +9, +15, +18, +21[cp4]/55, XXX, +1, +6, +7, +9, t(10;11) (p12;q14), +12, +15, +18, +21[1]/46,XX[1]
8	M/16	2.34/69/234	/	ALL/60.5	ALAL, CD7/33/99/117/cyCD3+	KRAS G12V, U2AF1 R35L, PHF6 c.968+2T>A	45–48, XY, t(10;11)(p12;q14) [cp2]/46, XY[8]
9	M/50	7/139/179	/	M1/92	AML, CD7/33/ 13/34/38/117+	SF3B1 G862L, WT1 R385fs, WT1 c.1113+1G>A, SUZ12 G511fs, ASXL3 P1253H, JAK3 A573V, JAK3 M511I, PHF6 H329R	46, XY, t(10;11)(p12;q14)[5]/46, XY[3]
10	F/34	18.9/99/159	Y	AL /85	ALAL, CD7/33/ 5/79a/22+	PHF6 L32Vfs*3, ASXL2 S924Lfs*21, ETV6 C338_Y344delinsFRA	45–46, XX, del(5)(q13q21), t(10;11)(p12;q14), del(13)(q21)[cp6]/46, XX[8]
11	F/27	2.7/96/152	/	M5/55.6	AML, CD7/33/ 117/34/DR+	NRAS A146Y, ARID1A V1817I, SRP72 Q546K	46, XX[20]
12	M/31	51/165/164	Y	ALL/89.2	B‐ALL, CD7/33/ 34/123/19/79a+	WT1 C458R, JAK3 M511I, PDGFRB R435C, IL7R T414M, BRCA2 N991D	43–49, XY, der(1)ins(1;?)(p22;?), +6, +7, +10, t(10;11)(p12;q14), +19[cp11]
13	M/32	4.3/157/270	Y	LBL/15	ETP‐ALL, CD7/ 33/5/cyCD3+	JAK3 L875H, CSMD1 G2504S	46, XY, t(10;11)(p12;q14) [1]/46, XY[14]
14	F/36	7.8/86/39	/	ALL /77.5	ETP‐ALL, CD7/ 33/34/13/cyCD3+	NRAS G12S, PHF6 C305F, RUNX1 A115V, JAK1 S703I, ARID1B A2129V	84–89, XXXX[cp3]/46, XX[4]
15	M/33	5.4/80/5	/	ALL/74.8	ETP‐ALL, CD7/ 33/34/38/99/cCD3/123/DR/5/56+	ASXL2 V785Sfs*14, IKZF1 R162Q, GATA2 R398W	46, XY, add(2)(q37), t(3;10)(p23;p15), del(11)(q23)/45–46, XY, t(3;10)(p23;p15), del(11)(q23), del(12)(p13)[cp13]
16	M/29	7.4/136/184	Y	AML/87.5	AML, CD7/33 /34/117/13/38/71/123/MPO/DR+	NOTCH1 Q2398Pfs*25, WT1 S386*, KRAS A59E, SUZ12 E314fs*3, PHF6 H329Q, DPF1 Y26F	81–91, XXYY, i(9q), t(10;11) (p12;q14) × 2[cp8]/46, XY[3]
17	M/26	167/105/155	Y	ALL/90	ETP‐ALL, CD7/ 33/34/38/56/99/DR/cyCD3+	NRAS A59T, EZH2 E194*, EZH2 c.1547‐2_1547‐1insCCTT, KDM6A N1103Mfs*32, PHF6 R319*	38–48, XY, +5, +6, i(7q), −10, del(11)(q23), +20[cp14]
18	M/30	1.65/91/192	/	AL/94	ALAL, CD7/33 /34/117/19/cCD79a/38/DR/71+	PHF6 Q308*, WT1 R375delinsPT*, U2AF1 R35L	87–96, XXXYYY, del(11)(q21)×2 [cp2]/46, XY, del(11)(q21)/46, XY[8]

Abbreviations: AL, acute leukemia; ALAL, acute leukemias of ambiguous lineage; ALL, acute lymphoblastic leukemia; AML, acute myeloid leukemia; EMD, extramedullary disease; FAB, French‐American‐British; G/A, gender/age; HB, hemoglobin; LBL, lymphoblastic lymphoma; PLT, platelet; WBC, white blood cell; Y, yes.

**FIGURE 1:**
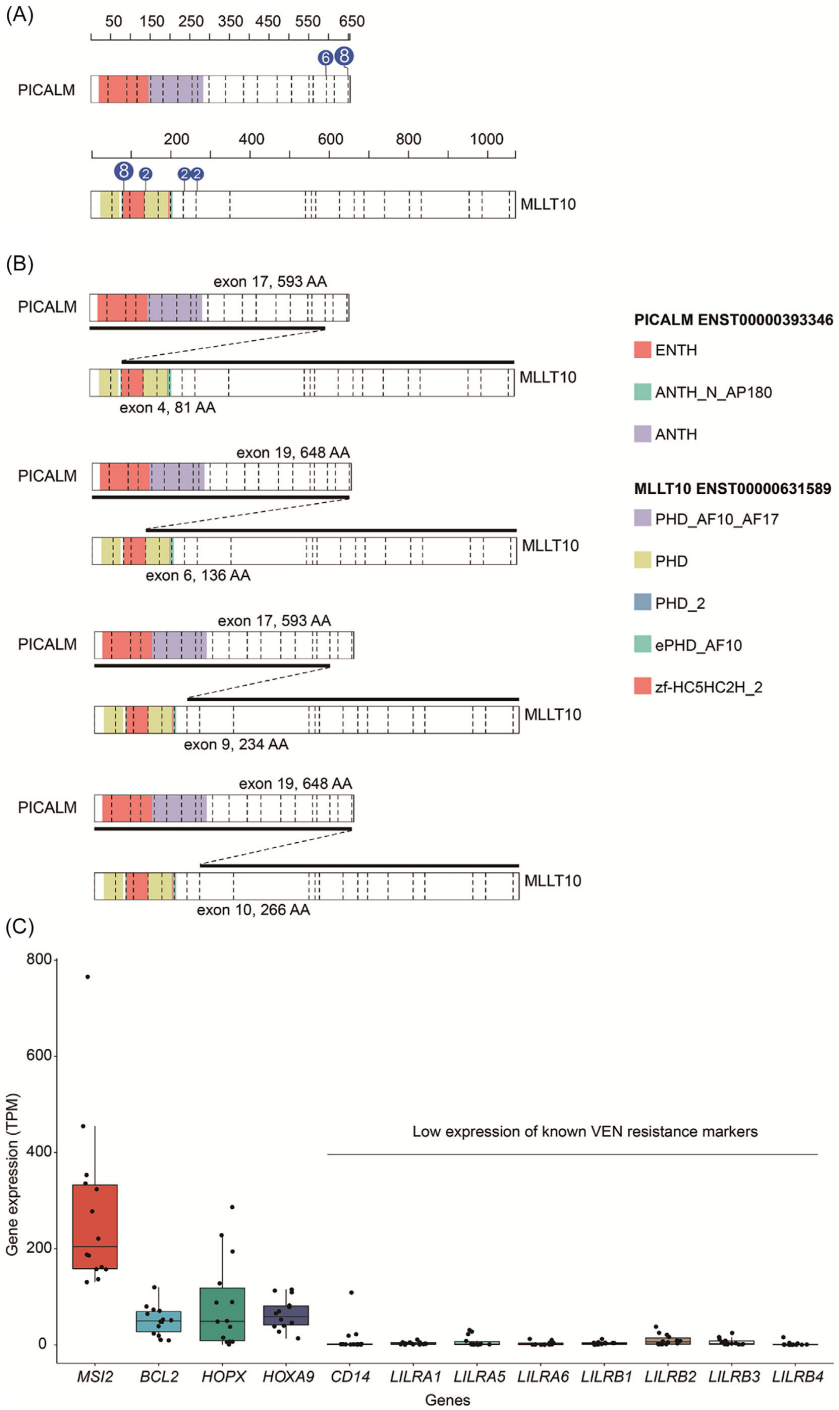
PICALM::MLLT10 fusion protein pattern diagram and expression of representative genes including BCL‐2, stemness genes, and genes associated with venetoclax (VEN) resistance in 14 *PICALM::MLLT10* positive patients. (A) The protein domains of PICALM and MLLT10, fusion gene breakpoints, and their corresponding patient numbers. The numbers inside the circle indicate the number of patients corresponding to that breakpoint fusion pattern. Among them, the *PICALM* fusion sites were mainly concentrated in exon 17 (*n* = 6) and exon 19 (*n* = 8). The *MLLT10* fusion site is most common with exon 4 (*n* = 8). (B) The breakpoints of *MLLT10* fusion site, corresponding to exon 4, exon 6, exon 9, and exon 10, respectively. (C) The ordinate is the expression level of the corresponding gene (transcripts per million (TPM) value is quantified). *MSI2* and *HOPX* are two known myeloid stemness genes (high expression in early hematopoietic stem cells). *HOXA9* is a target gene known to be regulated by PICALM::MLLT10. The CD14 and LILRA/B gene families are biomarkers (high expression in monocytes) known to be associated with VEN resistance. The results showed higher expression of BCL2 and myeloid stem genes but lower expression of VEN resistance‐related biomarkers in *PICALM::MLLT10* positive patients.

We further investigated the different fusion forms of *PICALM::MLLT10* and confirmed the corresponding broken fusion sites by sanger sequencing. The results showed that the *PICALM* breakpoints are mainly concentrated in exon 17 (*n* = 6) and exon 19 (*n* = 8). Exon 4 (*n* = 8), exon 6 (*n* = 2), exon 9 (*n* = 2), and exon 10 (*n* = 2) are the most common breakpoints of *MLLT10* (Figure 1A,B). Hence, this is the first report about the breakpoints and fusion gene forms of *PICALM::MLLT10*.

For initial treatment, eight patients including six T‐ALL, one B‐ALL, and one B/T MPAL received standard ALL induction chemotherapy (VDPCP, vincristine, idarubicin, pegaspargase, cyclophosphamide, prednisone), five patients first diagnosed as AML and ALAL received standard AML induction chemotherapy (3 + 7 IA regimen including idarubicin, cytarabine), two ALAL patients received standard AML induction chemotherapy combined with VP regimen (idarubicin, cytarabine, vincristine, prednisone), and one ETP‐ALL patient received Hyper‐CVAD regimen. It should be mentioned that one ETP‐ALL patient first received venetoclax combined with chemotherapy regimen including low doses of cytarabine (LDAC), granulocyte colony‐stimulating factor (G‐CSF), and homoharringtonine (HAG), one ALAL patient also first received venetoclax combined with azacytidine as well as VP regimen, and both achieved complete remission (CR; Patients 15 and 18, Table 2). Except these two patients, CR rate of the other 16 patients was only 31.3% (5/16), and 11 patients showing no remission (NR). These NR patients including five ALAL (5/5), four T‐ALL (4/6), one AML (1/2), and one B/T MPAL (1/1) subsequently received salvage chemotherapy. It is worth mentioning that five of them (5/11) received combined chemotherapy regimen including LDAC, G‐CSF, and anthracyclines such as aclarubicin or idarubicin or homoharringtonine (CAG or IAG or HAG), and two of five patients also further received combination therapy with venetoclax (CAG or IAG + VEN). Finally, all the total five patients dramatically achieved CR (100%, 5/5) after CAG or HAG alone (Patients 3, 8, and 9, Table 2) and CAG or IAG + VEN (Patients 2 and 10, Table 2 and Figure S1G). In addition, two patients including one AML and one ETP‐ALL also achieved CR after salvage venetoclax application (Patients 16 and 17, Table 2). In the remaining four patients, one patient who was failure to initial Hyper‐CVAD A/B regimen also dramatically achieved CR after the more Hyper‐CVAD A regimen combined with venetoclax (Patient 13, Table 2), one patient achieved CR while another still NR with both receiving CLAG (cladribine, cytarabine, G‐CSF) regimen (Patients 1 and 4, Table 2), and the B/T MPAL patient still achieve no any remission even after FLT3 inhibitor gilteritinib and blinatumomab application (Patient 7, Table 2). Subsequently 11 patients (nine CR, two with refractory disease) received allogenic hematopoietic stem cell transplantation (allo‐HSCT), and all the CR patients survived well after HSCT (follow‐up 3–24 months, median 15 months) without relapse, while two NR patients died soon after transplantation because of severe complications (Patients 1 and 7, Table 2).

**TABLE 2:** Clinical treatment and outcome of *PICALM::MLLT10* positive AL patients.

N	Immunophenotype	Induction	Response	Reinduction	Response	Status before HSCT	HSCT	Outcome, OS (m)
**1**	ALAL	IA	NR	CLAG	NR	NR	Y	Die, 3
**2**	ETP‐ALL	VDPCP	NR	CAG+VEN	CR	CR, MRD+	Y	Live, 22
**3**	ALAL	VDPCP	NR	CAG	CR	/	N	Die, 17
**4**	ALAL	IA	NR	CLAG	CR	CR, MRD+	Y	Live, 36
**5**	Cortical T‐ALL	VDPCP	CR	/	/	CR, MRD‐	Y	Live, 35
**6**	ETP‐ALL	VDPCP	CR	/	/	CR, MRD+	Y	Live, 35
**7**	B/T	VDPCP	NR	CVAD, Gil, Blincyto	NR	NR	Y	Die, 10
**8**	ALAL	IA+VP	NR	CAG	CR	CR	Y	Live, 28
**9**	AML	IA	NR	HAG	CR	/	N	Die, 12
**10**	ALAL	IA+VP	NR	IAG+VEN	CR	/	N	Live, 14
**11**	AML	IA	CR	/	/	CR	Y	Live, 29
**12**	B‐ALL	VDPCP	CR	/	/	CR, MRD+	Y	Live, 22
**13**	ETP‐ALL	CVAD/MA	NR	CVAD +VEN	CR	CR	Y	Live, 19
**14**	ETP‐ALL	VDPCP	CR	/	/	CR, MRD‐	Y	Live, 15
**15**	ETP‐ALL	HAG+VEN	CR	/	/	/	N	Live, 8
**16**	AML	IA	NR	AZA+VEN	CR	/	N	Live, 4
**17**	ETP‐ALL	VDPCP	NR	VEN	CR	/	N	Live, 4
**18**	ALAL	VP+AZA+VEN	CR	/	/	/	N	Live, 2

Abbreviations: AL, acute leukemia; ALAL, acute leukemias of ambiguous lineage; ALL, acute lymphoblastic leukemia; AML, acute myeloid leukemia; AZA, azacytidine; Blincyto, Blinatumomab; CAG, LDAC, aclarubicin and G‐CSF; CLAG, cladribine, high‐dose cytarabine and G‐CSF; CVAD/MA, hyper‐cyclophosphamide, vincristine, doxorubicin, and dexamethasone alternating with methotrexate/cytarabine; ETP‐ALL, early T‐cell precursor ALL; Gil, gilteritinib; HAG, LDAC, homoharringtonine and G‐CSF; HSCT, hematopoietic stem cell transplantation; IA, idarubicin and cytarabine; IAG, LDAC, idarubicin and G‐CSF; MRD, measurable residual disease; OS, overall survival; VDPCP, vincristine, idarubicin, cyclophosphamide, pegaspargase, and prednisone; VEN, venetoclax.

## 3 DISCUSSION

In our patients’ series, we have shown that *PICALM::MLLT10* positive AL is associated with unique biological characteristic such as miscellaneous immunophenotype including T‐ALL, ALAL, AML, and B‐ALL, complex karyotype and frequently concomitant PHF6 mutation. In addition, EMD can be found in half of the total patients. In terms of treatment, this patient group was found to be shown poor initial treatment response to standard chemotherapy but sensitivity to combining chemotherapy especially with venetoclax introduction. We further analyzed the possible treatment mechanism of venetoclax in this patient group and found that BCL‐2 itself was highly expressed, indicating that BCL‐2 inhibitor should be effective. While *MSI2* and *HOPX* as two known myeloid stemness genes were shown high expression, *HOXA9* as target gene directly regulated by *PICALM::MLLT10* was also shown high expression, which further indicated that these patients with an earlier leukemic stem cell phenotype can benefit from venetoclax [10]. In addition, the lower expression of CD14 and LILRA/B family as biomarkers of venetoclax resistance also verified the efficacy of venetoclax in this subgroup patients [11] (Figure 1C).

It has already been reported that the prognosis of *PICALM::MLLT10* positive T‐ALL depends on the stage of leukemia cell maturation arrest. Mature TCRγδ +* PICALM::MLLT10* positive T‐ALL responds well to standard treatment, whereas TCR‐*PICALM::MLLT10* positive T‐ALL has a strikingly inferior outcome [1], suggesting that an earlier stage of leukemia stem cells (LSCs) is insensitive and resistant to conventional therapy. In our study, these patients showed poor response to initial AML or ALL‐like chemotherapy, but especially sensitive to venetoclax as well as LDAC, G‐CSF, and anthracyclines combination chemotherapy despite the complexity of immunophenotyping and cytogenetics at the first diagnosis. One possible reason may be that *PICALM::MLLT10* fusion occur in the early stage of hemopoietic stem cells. Dutta et al. showed that the cell of origin of leukemia with *PICALM::MLLT10* is stem or very early multipotent cell, but not B‐cell lineage with a phenotype similar to LSC [12]. G‐CSF can prime G0/G1 phase LSC into the S phase, which may help the leukemia cells sensitivity to LDAC and anthracyclines [13, 14]. The above study was also align with our findings. In addition, venetoclax had been reported eradicating LSCs of AML patients by disrupting the mechanisms of cellular energy metabolism [15].

On the other hand, PHF6 and JAK3 mutations have been confirmed to cooperate and drive T‐ALL progression [16], which is consistent with our study showing PHF6 and JAK3 mutations ranking 1 and 2 individually. Furthermore, *PICALM::MLLT10* positive AL is clinically characterized by extramedullary involvement [4], also as showing in our study, indicating more aggressive and refractory clinical features. If extramedullary lesion is persisting, the subsequent allo‐HSCT will be delay even though measurable residual disease (MRD) of bone marrow is negative. Moreover, CD7 chimeric antigen receptor (CAR)‐T cell [17] or gemtuzumab ozogamicin (GO) may also serve as salvage therapy for patients especially failure of LDAC, G‐CSF, and anthracyclines combination chemotherapy as well as venetoclax, but all of these speculations need more clinical data.

Taken together, our data suggested *PICALM::MLLT10* positive AL showing miscellaneous immunophenotype, higher expression of leukemic stemness genes and lower expression of biomarkers of venetoclax resistance, more extramedullary involvement, and especially poor response to conventional induction chemotherapy which is alongside with [18], but may benefit from venetoclax as well as LDAC, G‐CSF, and anthracyclines combination chemotherapy. Sequential HSCT after chemotherapy combined with venetoclax may further improve long‐term survival in AL patients with CR even MRD positive.

## AUTHOR CONTRIBUTIONS

Yongmei Zhu, Jianfeng Li, and Lingling Zhao performed RNA‐seq and NGS analysis. Haimin Sun and Zeying Yan were in charge of patient treatment. Guang Yang performed cytogenetic analysis. Haimin Sun and Sujiang Zhang collected and analyzed data as well as wrote the manuscript, Sujiang Zhang designed and supervised the whole study. All authors reviewed and approved the final manuscript.

## CONFLICTS OF INTEREST STATEMENT

The authors declare no conflicts of interest.

## ETHICS STATEMENT

The authors have confirmed ethical approval statement is not needed for this submission.

## PATIENT CONSENT STATEMENT

The authors have confirmed patient consent statement is not needed for this submission.

## CLINICAL TRIAL REGISTRATION

The authors have confirmed clinical trial registration is not needed for this submission.

## Supporting information

Supporting Information

## Data Availability

Available RNA sequencing data of patients are available on GSA‐Human database (https://ngdc.cncb.ac.cn/gsa‐human/) under the accession number HRA004939. Other data that support the findings of this study are available from the corresponding author upon reasonable request.
