# Supplementary material for: PICALM::MLLT10 may indicate a new subgroup of acute leukemias with miscellaneous immunophenotype and poor initial treatment response but showing sensitivity to venetoclax
Source: EJHaem. 2024 May 15;5(3):565–72. doi: 10.1002/jha2.922 (PMC11182389; doi:10.1002/jha2.922)
Supplement: Supplementary file 1 — Supporting Information [file JHA2-5-565-s001.pdf]

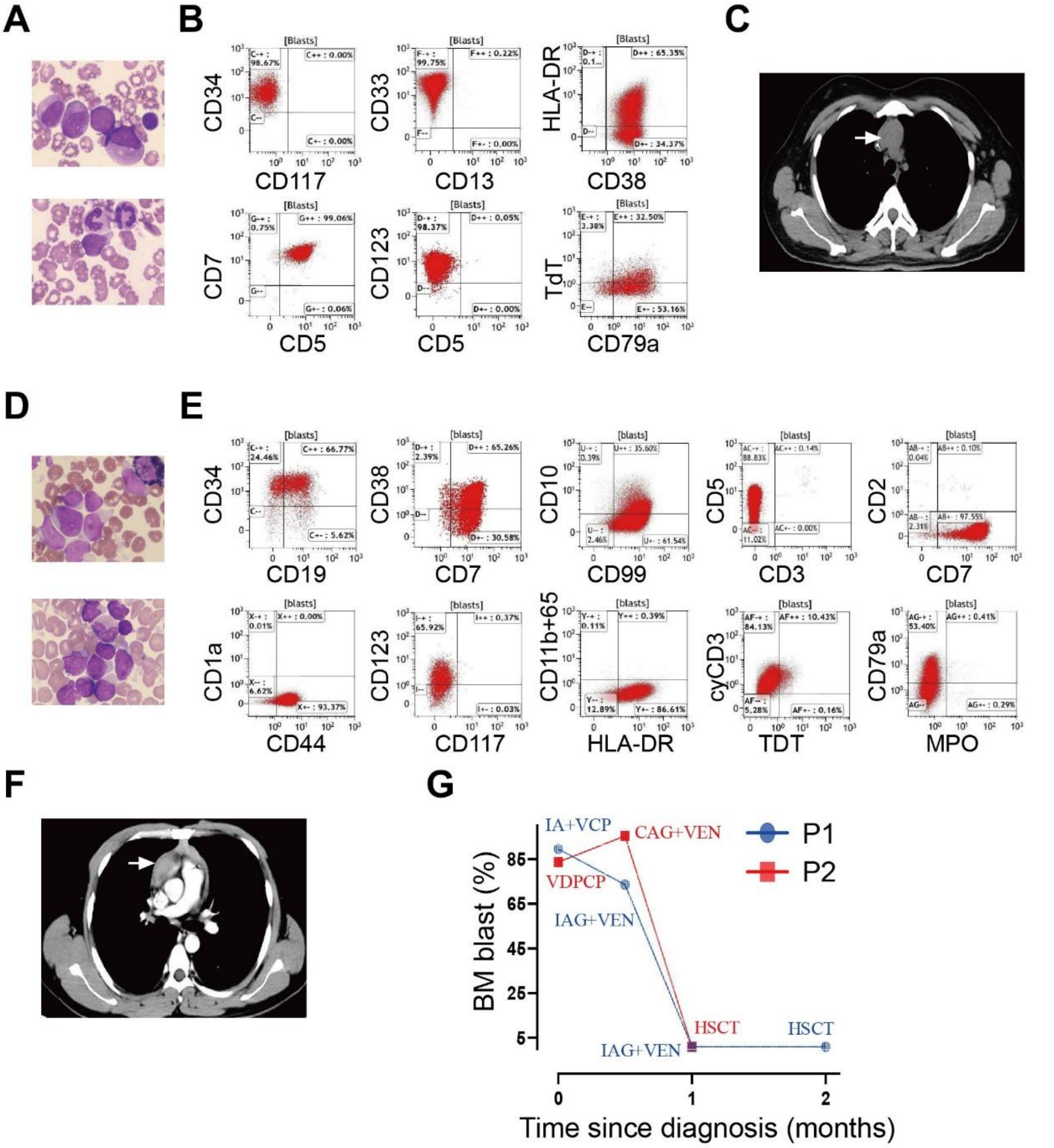

Supplementary Figure 1. Clinical data of *PICALM::MLLT10* positive acute leukemia patients. (A & D) Typical bone marrow morphology in two patients. (B & E) Flow cytometry showing typical immunophenotype of acute leukemias of ambiguous lineage of these two patients. (C & F) Chest CT images showing significantly enlarged mediastinum in these two patients. (G) The two patients showed poor response to initial AML or ALL-like chemotherapy, but both achieved CR after chemotherapy regimen including LDAC, G-CSF and anthracyclines combined with venetoclax (IAG or CAG +VEN).
